# Atrial fibrillation episode status and incidence of coronary slow flow: A propensity score-matched analysis

**DOI:** 10.3389/fcvm.2023.1047748

**Published:** 2023-03-20

**Authors:** Ya-fang Gao, Yan Chen, Cheng-jian Wang, Ying Du, Ya-hui Ding

**Affiliations:** ^1^ Heart Center, Department of Cardiovascular Medicine, Zhejiang Provincial People's Hospital (Affiliated People's Hospital, Hangzhou Medical College), Hangzhou, China; ^2^ Graduate Department, Bengbu Medical College, Bengbu, China; ^3^ The Second Clinical Medical College, Zhejiang Chinese Medical University, Hangzhou, China

**Keywords:** coronary slow flow, atrial fibrillation, corrected TIMI frame count, cardiac rhythm, propensity score matching

## Abstract

**Background:**

Previous studies have shown that patients with a history of atrial fibrillation (AF) have a higher risk of developing coronary slow flow (CSF). However, whether AF episode status affects the incidence of CSF has not been confirmed. This study investigated the correlation between AF episode status and the incidence of CSF.

**Methods:**

We enrolled patients with AF who underwent coronary angiography for symptoms of myocardial ischemia between January 1, 2017, and April 30, 2022, at our institution and classified them according to whether they had an episode of AF in the perioperative period. The outcomes were defined the occurrence of CSF overall and in each of the three coronary arteries. The analysis was repeated after adjusting the baseline information by the propensity score matching method in a 1:1 ratio.

**Results:**

214 patients who met the inclusion and exclusion criteria were included in the study (AF episode group: 100 patients, AF non-episode group: 114 patients). Before matching, age, left atrial size, ejection fraction, heart rate, CSF incidence, and mean corrected thrombolysis in myocardial infarction frame counts were higher in patients with intraoperative AF episodes than in patients without episodes. To prevent the dependent variable (CSF incidence) from being confounded by confounding factors, we matched the two groups for age, left atrial size, and ejection fraction. In the logistic regression analysis, the incidence of CSF was significantly higher in the intraoperative AF episode group (*P* = 0.010, OR = 2.327, 95% CI: 1.226–4.416) than in the non-episode group.

**Conclusion:**

In patients with AF, AF episode status is significantly correlated with an increased overall incidence of CSF.

## 1. Introduction

Atrial fibrillation (AF) is the most common arrhythmia in clinical practice and is characterized by impaired atrial excitation leading to the deterioration of atrial function (1, 2). According to the 2017 Global Burden of Disease Study, there were 37.6 million persons with AF globally, including 3 million people with new-onset AF and 287,000 people who died from AF (3). AF causes various disabling symptoms, including blood pressure drops, palpitations, poor exercise tolerance, and lung congestion (4). Furthermore, even in the absence of severe coronary artery disease, patients with AF may experience symptoms caused by changes in myocardial perfusion and brief myocardial ischemia such as angina-like chest pain (5, 6).

Coronary slow flow (CSF) is a specific phenomenon of coronary microcirculatory dysfunction which is characteristic of delayed distal perfusion of the vessel in the absence of significant epicardial coronary stenosis (7). It may also be referred to as ischemia with non-obstructive coronary arteries (8). More than 80% of patients endure repeated bouts of resting angina, and over 20% require hospitalization for associated treatments such as intravenous nitrates (9). Thus far, the pathophysiological mechanisms underpinning CSF and its impact on cardiac function remain unclear. Its causative factors may include endothelial and microvascular dysfunction, small vessel breakdown, inflammation, diffuse atherosclerosis, platelet dysfunction, and impaired lipid metabolism (10, 11). This suggests CSF and AF may have similar risk factors (12, 13). A previous report has demonstrated a correlation between a history of AF and CSF (14). Some animal studies have shown that inducing an acute episode of AF affects coronary perfusion and coronary flow reserve (15). However, whether the two are correlated in humans in the non-acute phase needs further confirmation. This study is the first to explore the relationship between AF episode status and CSF using corrected thrombolysis in myocardial infarction (TIMI) frame counts (CTFC) in patients.

## 2. Methods

### 2.1. Study design and population

In this retrospective cohort study, we included patients with AF who underwent coronary angiography for symptoms of myocardial ischemia (including chest tightness, angina, and palpitations) (5) from January 1, 2017, to April 30, 2022, at Zhejiang Provincial People's Hospital. The classification was based on whether AF persisted in the perioperative period in the nursing record (data from bedside monitor during hospitalization). The exclusion criteria were as follows: patients with a history of coronary revascularization (Percutaneous coronary intervention/coronary artery bypass graft), coronary stenosis more than or equal to 50%, severe heart failure (New York Heart Association class III-IV), severe atrioventricular block, severe valvular heart disease, congenital heart disease, active autoimmune disease, severe renal failure, and malignancy. After obtaining a detailed medical history, all patients underwent a thorough physical examination. The protocol for this study was approved by the Ethics Committee of Zhejiang Provincial People's Hospital.

### 2.2. Data collection

The following information was collected from the electronic medical record system of patients who met the inclusion criteria: general clinical data (sex, age, height, weight, body mass index), personal history (hypertension, history of smoking), cardiac ultrasound indices (left atrial size, ejection fraction), and laboratory indices (creatinine, uric acid, hemoglobin A1c, homocysteine, total cholesterol, low-density lipoprotein cholesterol, high-density lipoprotein cholesterol, troponin I, B-type natriuretic peptide, prothrombin time, activated partial thromboplastin time, thrombin time, D-dimer). We observed the coronary angiography videos of all the patients and analyzed them with the procedure and nursing records to determine whether the patients had AF episodes. Moreover, we collected the intraoperative mean heart rate, systolic blood pressure, diastolic blood pressure, and overall CSF occurrence, in each patient as well as each coronary artery's diameter, myocardial bridge, and CSF occurrence.

### 2.3. Coronary angiography and CTFC

**Figure 1:**
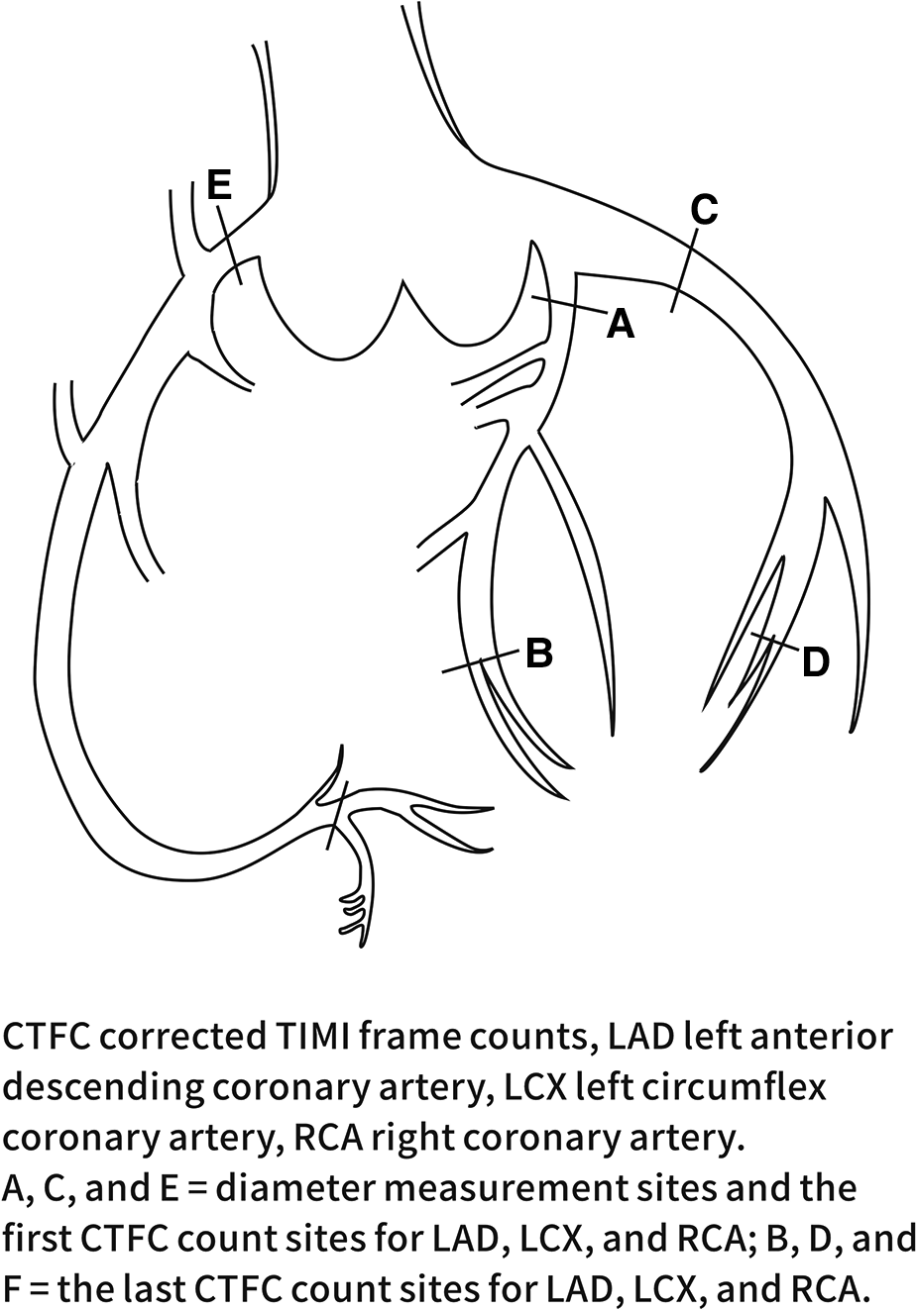
Diameter measurement sites and CTFC count sites for each coronary artery.

This study defined CSF according to the TIMI test and the CTFC method. All angiograms were performed using the standard Judkins technique (16) and evaluated by two experienced cardiologists. The first frame was defined as the frame in which the contrast agent contacted the medial wall of the coronary artery, filled more than 70% of its diameter, and advanced steadily. The last frame was defined as the frame where the leading edge of the contrast agent reached the end of the coronary artery branch. The specific frame positioning is shown in Figure 1. The frame count in the left anterior descending coronary artery (LAD) was divided by a factor of 1.7 to correct for its longer length. Internationally, coronary angiography is performed at 30 frames per second (17). In this study, the recorded frame rate was 15 frames per second, so the number of structures obtained was multiplied by 2.

According to Gibson et al. a mean CTFC of more than 27 frames in three coronary arteries was defined as CSF. The CTFC cut-off values for each coronary artery were 21.3 ± 1.5 frames for the LAD, 22.2 ± 4.1 frames for the left circumflex coronary artery (LCX), and 20.4 ± 3 frames for the right coronary artery (RCA). When the CTFC was more than 2 standard deviations of these means then CSF was said to occur in those coronary arteries (16).

### 2.4. Measurement of coronary artery diameter

The diameter of each coronary artery in the coronary angiography video was measured by two trained independent observers using the MicroDicom DICOM viewer 3.4.7 under clinical data confidentiality. The widest dimension of the proximal coronary artery was measured in diastole according to the methodology of Lip et al. (18). The diameter of the coronary artery was assessed using a known catheter tip diameter (1.67 mm for a 5.0 F catheter) as a calibration object with the image intensifier settings held constant. The LAD and the LCX were measured at a 30° right anterior oblique projection and the RCA was measured at a 60° left anterior oblique projection. The specific position is shown in Figure 1.

### 2.5. Statistical analysis

The data was analyzed using IBM SPSS Statistics for Windows, version 26.0 (IBM Corp., Armonk, N.Y., United States) and R software version 4.2.1. We grouped patients according to whether they were in an AF episode state during the perioperative period. Patients in different groups were matched in a 1:1 ratio using propensity score matching (PSM) to reduce baseline differences according to the methodology of the study by Teoh AYB et al. (19, 20). We calculated a propensity score of 0.1 for maximum execution performance and fixed caliper width. The measurement data was expressed as means ± standard deviations or medians (interquartile range), and the difference between the means of the two groups were tested by the Student *t* test or nonparametric tests. Categorical data was expressed using percentages, and differences between the two groups were compared using the chi-square test. Before and after PSM, factors associated with intraoperative AF episode status were analyzed using logistic regression analyses. After matching was completed, standardized mean differences (SMD) evaluated the balance of covariates. All tests were two-tailed, and a *P* value of less than 0.05 was considered to be statistically significant.

## 3. Results

### 3.1. Demographic and clinical characteristics

**Figure 2:**
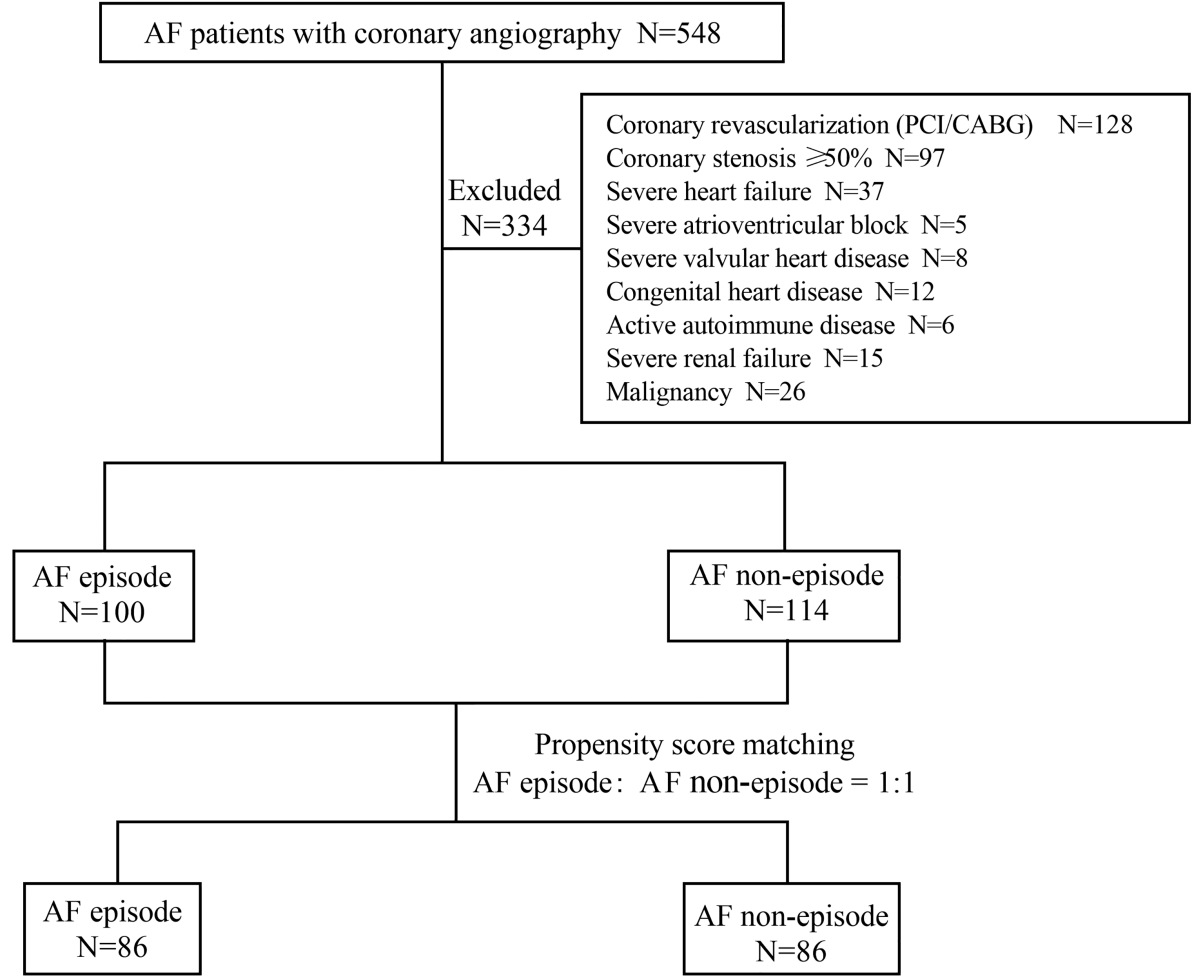
Patient screening process.

Between January 1, 2017, and April 30, 2022, 548 patients with AF underwent coronary angiography in our hospital due to symptoms of myocardial ischemia. 214 patients met the inclusion and exclusion criteria (Figure 2). These included 100 patients with persistent episodes of AF in the perioperative period (46.73%) and 114 patients with a history of AF but who did not currently have AF (53.2%). Before PSM, patients in the AF episode group were older than those in the non-episode group (72.71 ± 8.61 years vs. 68.89 ± 9.10 years, *P* = 0.002) and had a larger left atrial size [43.0 (8.00) mm vs. 40.0 (9.00) mm, *P* = 0.015] and ejection fraction [64.0 (6.00) % vs. 61.5 (10.00) %, *P* = 0.011]. Other general clinical data (sex, height, weight, body mass index), personal history (hypertension, smoking), and laboratory parameters (creatinine, uric acid, Hemoglobin A1c, homocysteine, total cholesterol, low-density lipoprotein cholesterol, high-density lipoprotein cholesterol, troponin I, B-type natriuretic peptide, prothrombin time, activated partial thromboplastin time, thrombin time, and D-dimer) were not statistically significant in both groups (Table 1).

**Table 1:** Baseline characteristics of patients with and without AF episodes before PSM.

Characteristics	Non-intraoperative AF episode (*n* = 114)	Intraoperative AF episode (*n* = 100)	SMD	*P* value
Age (years)	68.89 ± 9.10	72.71 ± 8.61	0.433	0.002
Sex (male), *n* (%)	60 (52.63%)	53 (53.00%)	0.135	0.957
BMI (kg/m^2^)	24.17 ± 3.09	24.54 ± 4.88	0.137	0.425
Height (cm)	164.5 (12.00)	163.0 (12.00)	−0.051	0.782
Waistline (kg)	65.0 (16.50)	68.5 (17.50)	0.074	0.436
Hypertension, *n* (%)	70 (61.40%)	58 (58.00%)	−0.241	0.612
Smoking, *n* (%)	33 (28.95%)	31 (31.00%)	−0.248	0.744
**Echocardiography parameters**				
LVEF (%)	61.5 (10.00)	64.0 (6.00)	0.282	0.011
LA (mm)	40.0 (9.00)	43.0 (8.00)	0.273	0.015
**Laboratory's test**				
Creatinine (μmol/L)	76.75 (23.30)	80.45 (24.90)	0.067	0.165
Uric acid (μmol/L)	357.5 (141.00)	354.0 (153.00)	0.077	0.821
HbA1c (%)	6.15 (1.93)	5.95 (1.38)	−0.241	0.128
TC (mmol/L)	3.98 ± 1.34	3.92 ± 1.48	−0.068	0.677
LDL-C (mmol/L)	2.31 ± 0.77	2.40 ± 0.80	0.114	0.378
HDL-C (mmol/L)	1.08 ± 0.27	1.07 ± 0.30	−0.035	0.928
cTnI (µg/L)	0.006 (0.013)	0.005 (0.009)	−0.137	0.407
BNP (pg/ml)	104.75 (172.35)	134.35 (217.40)	0.226	0.153
PT (s)	11.4 (1.10)	11.4 (1.20)	−0.059	0.758
APTT (s)	27.0 (3.80)	26.6 (4.00)	−0.056	0.158
TT (s)	17.65 (1.60)	17.7 (1.60)	0.160	0.729
D-Dimer (µg/L)	300.0 (455.00)	375.0 (470.00)	0.146	0.203

BMI, body mass index; LVEF, ejection fraction; LA, left atrial size; HbA1c, glycosylated hemoglobin; TC, total cholesterol; LDL-C, low density lipoprotein cholesterol; HDL-C, high density lipoprotein cholesterol; cTnI, cardiac troponin I; BNP, brain natriuretic peptide; PT, prothrombin time; APTT, activated partial thromboplastin time; TT, thrombin time.

To balance the clinical baseline data, we matched for age, left atrial size, and ejection fraction (data from cardiac ultrasound). 172 patients were included in this study's experimental and control groups in a 1:1 ratio. 42 patients were excluded because they were not successfully matched. Baseline information was not statistically significant in either group after PSM (Table 2).

**Table 2:** Baseline characteristics of patients with and without AF episodes after PSM.

Characteristics	Non-intraoperative AF episode (*n* = 86)	Intraoperative AF episode (*n* = 86)	SMD	*P* value
Age (years)	70.94 ± 8.62	71.77 ± 8.56	0.098	0.529
Sex (male), *n* (%)	45 (52.33%)	51 (59.30%)	0.142	0.357
BMI (kg/m^2^)	24.15 ± 2.93	24.44 ± 3.80	0.117	0.576
Height (cm)	165.0 (12.00)	164.5 (12.00)	−0.117	0.521
Waistline (kg)	66.06 ± 10.57	65.98 ± 12.81	0.014	0.964
Hypertension, *n* (%)	54 (62.79%)	49 (56.98%)	−0.117	0.437
Smoking, *n* (%)	26 (30.23%)	29 (33.72%)	0.074	0.624
**Echocardiography parameters**				
LVEF (%)	61.5 (10.00)	63.0 (7.00)	0.131	0.178
LA (mm)	41.0 (10.00)	43.0 (10.00)	0.096	0.336
**Laboratory's test**				
Creatinine (μmol/L)	78.15 (23.4)	80.90 (23.0)	−0.010	0.276
Uric acid (μmol/L)	367.5 (141.0)	353.0 (142.0)	0.059	0.938
HbA1c (%)	6.3 (1.85)	5.9 (1.25)	0.000	0.054
TC (mmol/L)	4.01 ± 0.94	3.95 ± 1.13	−0.067	0.679
LDL-C (mmol/L)	2.35 ± 0.77	2.42 ± 0.82	0.087	0.539
HDL-C (mmol/L)	1.08 ± 0.30	1.08 ± 0.43	−0.036	0.998
cTnI (µg/L)	0.005 (0.013)	0.005 (0.008)	−0.157	0.595
BNP (pg/ml)	114.3 (177.3)	139.4 (241.0)	0.192	0.268
PT (s)	11.45 (1.15)	11.40 (1.22)	−0.060	0.470
APTT (s)	26.6 (3.70)	26.5 (4.70)	0.006	0.606
TT (s)	17.6 (1.40)	17.7 (1.70)	0.158	0.830
D-Dimer (µg/L)	300.0 (465.00)	365.0 (572.50)	0.183	0.339

BMI, body mass index; LVEF, ejection fraction; LA, left atrial size; HbA1c, glycosylated hemoglobin; TC, total cholesterol; LDL-C, low density lipoprotein cholesterol; HDL-C, high density lipoprotein cholesterol; cTnI, cardiac troponin I; BNP, brain natriuretic peptide; PT, prothrombin time; APTT, activated partial thromboplastin time; TT, thrombin time.

### 3.2. Outcomes

**Figure 3:**
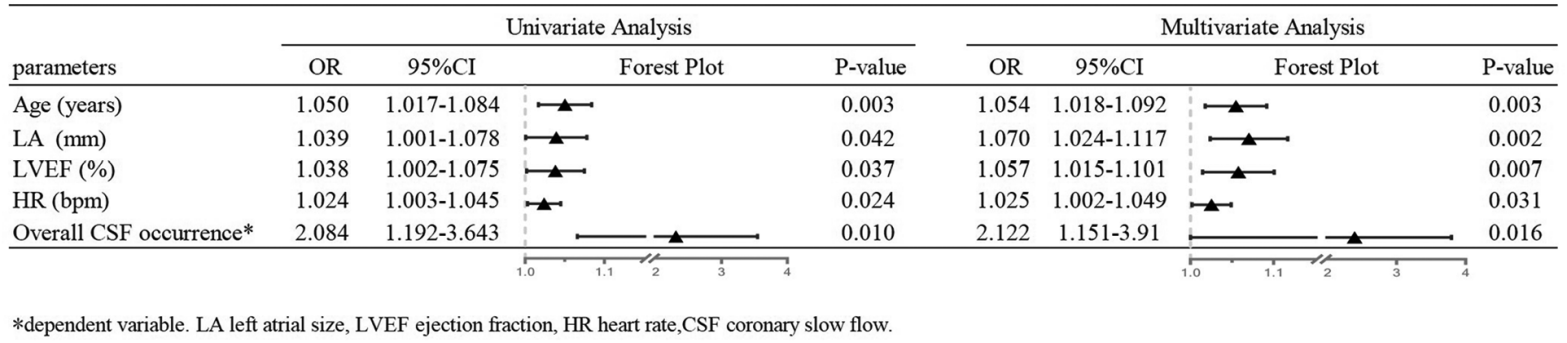
Univariate and multifactor logistic regression before PSM matching.

**Figure 4:**
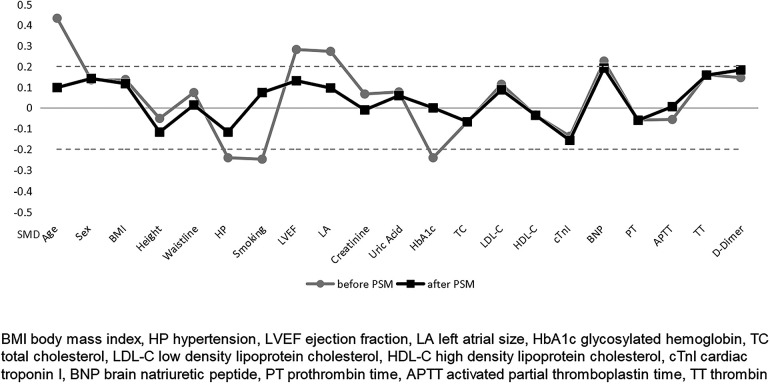
Balance of baseline information before and after PSM matching in two groups.

**Figure 5:**
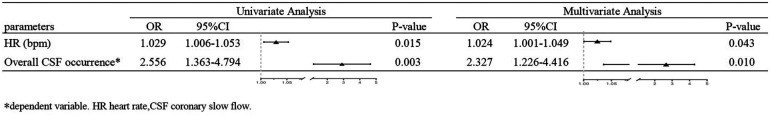
Univariate and multifactor logistic regression after PSM matching.

The primary outcome indicators for this study were overall CSF occurrence and mean CTFC, while secondary outcome indicators were CSF occurrence and mean CTFC of each coronary artery. Before PSM, age, left atrial size, ejection fraction, mean intraoperative heart rate, and overall CSF incidence in the intraoperative AF group was statistically significant between the two groups (Tables 1, 3, *P* < 0.05). We, therefore, included these factors in a multifactorial logistic regression analysis and found that their statistical significance remained highly significant (Figure 3). The overall incidence of CSF (*P* = 0.016, OR = 2.122, 95% CI: 1.151–3.910) was higher in the AF episode group compared with the control group. To exclude confounding factors for the dependent variable (overall CSF occurrence), we matched age, left atrial size, and ejection fraction in both groups. The final baseline information obtained for both groups was balanced (Table 2, SMD < 0.02, Figure 4). Because of the high heart rate of patients with AF episodes, forced matching would result in many samples being excluded. Therefore, we kept the heart rate in the multifactorial logistic regression model to reduce the interference with the overall CSF incidence analysis results according to the methodology of Sulaiman et al. (21–23). The results showed that the overall incidence of CSF was significantly higher in the intraoperative AF episode group (*P* = 0.010, OR = 2.327, 95% CI: 1.226–4.416) than in the non-episode group (Figure 5). However, the differences in mean systolic and diastolic blood pressure between the two groups were not statistically significant, regardless of whether they were matched. The diameters, the incidence of myocardial bridges, CSF, and the occurrence of CTFC in the three coronary arteries were not significantly different between the two groups (Table 3). Therefore, our results were not affected by intraoperative patient blood pressure, coronary artery diameter, heart rate, or myocardial bridges.

**Table 3:** CTFC, diameter and heart rate in two groups of patients before and after PSM in coronary angiography.

Vessel	Before propensity score (PS) matching			After propensity score (PS) matching		
	Non-intraoperative AF episode (*n* = 114)	Intraoperative AF episode (*n* = 100)	*P* value	Non-intraoperative AF episode (*n* = 86)	Intraoperative AF episode (*n* = 86)	*P* value
HR (bpm)	78.0 (17.44)	82.44 (22.65)	0.051	77.0 (15.00)	82.4 (24.00)	0.032
SBP (mmHg)	132 (24.00)	130 (26.00)	0.749	133 (27.00)	130 (24.00)	0.800
DBP (mmHg)	77 (14.00)	79 (16.00)	0.774	77 (15.00)	80 (16.00)	0.560
**CTFC**						
LAD	22.35 (8.18)	24.00 (8.75)	0.061	22.35 (7.29)	24.35 (9.40)	0.122
LCX	26.00 (12.00)	26.00 (12.00)	0.098	26.00 (12.00)	26.00 (12.00)	0.159
RCA	24.00 (12.00)	24.00 (13.00)	0.336	24.00 (11.00)	25.50 (12.00)	0.107
Mean	23.55 (8.00)	25.49 (7.33)	0.025	23.55 (7.62)	27.43 (7.39)	0.018
**CSF occurrence**						
LAD	41 (35.96%)	48 (48.00%)	0.075	32 (37.21%)	43 (50.00%)	0.091
LCX	38 (33.33%)	38 (38.00%)	0.477	28 (32.56%)	32 (37.21%)	0.522
LCX	47 (41.23%)	43 (43.00%)	0.793	32 (37.21%)	41 (47.67%)	0.165
Overall	35 (30.70%)	48 (48.00%)	0.010	25 (29.07%)	44 (51.16%)	0.003
**Diameter**						
LAD	3.42 (0.68)	3.48 (0.77)	0.502	3.42 (0.69)	3.48 (0.69)	0.803
LCX	3.33 (0.62)	3.46 (0.71)	0.359	3.39 (0.58)	3.47 (0.70)	0.538
RCA	3.48 (0.57)	3.45 (0.80)	0.615	3.50 (0.66)	3.45 (0.75)	0.828
**Myocardial bridges**						
LAD	11 (9.65%)	12 (12.00%)	0.580	11 (12.79%)	12 (13.95%)	0.823
LCX	0	0	-	0	0	-
RCA	0	0	-	0	0	-

CTFC, corrected TIMI frame counts; HR, heart rate; SBP, systolic blood pressure; DBP, diastolic blood pressure; LAD, left anterior descending coronary artery; LCX, left circumflex coronary artery; RCA, right coronary artery; CSF, coronary slow flow.

## 4. Discussion

Previous studies have shown that patients with a history of AF have a higher incidence of CSF (24). This finding was also confirmed by Zhuang et al. in a retrospective study that included 2,060 patients with chest pain and no significant coronary stenosis and assessed coronary blood flow velocity using CTFC (14). Luo et al. concluded that the longer the duration of AF or the more extensive the left atrial diameter, the higher the CTFC in patients with AF. The mean CTFC independently predicted adverse cardiovascular events in patients with AF without the presence of coronary artery disease (25). AF may contribute to the progression of CSF through long-acting factors such as endothelial dysfunction (26, 27), chronic inflammation (28, 29), and atherosclerosis (30, 31); moreover, ventricular fibrosis as an irreversible long-acting factor may also play a significant role in this pathology (32–34). Several histopathological studies, using anti-CD34 antibodies, have shown that reduced microvessel density is associated with more severe fibrosis and microvessel density is lower in patients with AF compared with controls (35). Ventricular arrhythmias or reduced microvascular density may contribute to the development of CSF in patients with AF (24).

Based on the presentation and duration of AF, AF is commonly classified into four subtypes: paroxysmal, persistent, long-term persistent, and permanent (36). In experiments using dogs as a sample, Saito D et al. found that induction of acute AF resulted in a mild increase in coronary blood flow, a decrease in coronary vascular resistance, and a severe impairment of coronary flow reserve (15). Kochiadakis et al. found in a sample of 16 induced specific heart rates that the above findings for animals also apply to humans (37). Concurrently, the rapid, irregular contraction of atrial myocytes’ during AF episodes may contribute significantly to atrial energy expenditure without any practical external work (38). A significant increase in left atrial arteriovenous lactate was found during acute AF (39) which suggests that during acute AF, the increased demand for oxygen by the atria exceeds the supply. Such a mismatch between supply and demand could be responsible for the rapid decrease in atrial contractility reported 5 min after AF in goats (40) and 1 h after AF in pigs (41). In a pig model, gene expression profiles show changes in energy depletion 7 h after the onset of AF which may cause the process that triggers slow atrial structural remodeling (42). Structural remodeling occurs in patients with AF and goat AF models consistent with chronic ischemia and strongly reminiscent of hibernating ventricular myocardium due to chronic low-flow ischemia (43).

In this study, AF events were defined as those being in a continuous state of AF during the perioperative period rather than as acute occurrences. During the compensatory phase of a sustained AF episode, atrial remodeling decreases the energy expenditure and oxygen demand of the atria. Electrical remodeling, especially the reduction of calcium currents, strongly reduces the contractility of the atria (44). Range et al. used positron emission tomography to quantify myocardial perfusion in patients with persistent AF for the first time (45). The results found that patients with persistent AF had approximately 20% less myocardial blood flow in the resting state, 40% less in the congested state, and 22% less congestive blood flow reserve. The minimum coronary vascular resistance in patients with AF was significantly increased by approximately 62% compared with controls. One study found that myocardial blood flow perfusion was significantly lower in patients with AF than in controls before radiofrequency ablation, and that blood flow recovered after catheter ablation (restoration of sinus rhythm) (46). This study suggests that the AF episode is the primary cause for the myocardial perfusion flow abnormalities. Therefore, we suggest that an imbalance in myocardial supply and demand after an AF episode, with ongoing cumulative effects, promotes adaptation and various structural remodeling that ultimately contribute to CSF. As the above examples illustrate, these changes are reversible.

Furthermore, it has been suggested that the decrease in coronary blood flow may be related more to the change in RR interval. Kochiadakis et al. (37) found a significantly lower increase in coronary flow velocity integral per minute after AF induced by right atrial pacing compared with right atrial pacing with similar ventricular rates. The study also found no significant change in coronary flow velocity integral per minute after induced AF in 12 patients with complete AV block pacemakers with regular RR intervals. Irregularities in ventricular rhythm during atrial fibrillation can adversely affect the increase in coronary blood flow velocity. AF episodes result in increased short RR or long RR intervals. When the RR interval is significantly shortened, the left ventricular filling time is insufficient, and the cardiac output per beat is reduced, resulting in inadequate coronary artery supply or slowed blood flow (47). Moreover, a significant shortening of the diastolic phase can negatively affect diastolic-dominated coronary perfusion. When the RR interval is too long, it causes a significant decrease in coronary perfusion pressure in the late diastolic phase which also affects coronary perfusion (48). Therefore, we believe that this disordered rhythmic feature of AF may be one of the main causes of CSF. One study found that an increased ventricular rate caused severe changes in the waveform and amplitude of coronary blood flow velocity, delayed peak diastole, and significantly reduced blood flow by constructing a model mimicking the characteristics of heartbeats’ (49). During AF episodes, elevated heart rates and altered hemodynamic variability combine to cause coronary perfusion injury. When the ventricular rate is high, AF leads directly to reduced coronary microvascular flow (50).

This study has several limitations. First, coronary angiography cannot quantitatively assess the degree of vessel tortuosity and calcification. Subjective judgments are biased; hence, we did not use data in this regard. Second, our center only assesses the flow reserve fraction in patients with stenosis greater than 50% and lacks data on intraoperative coronary flow reserve. Third, our sample size could not be further expanded, and this study should be considered a pilot report.

## 5. Conclusion

Our study shows that in patients with AF, AF episode status is significantly correlated with an increased overall incidence of CSF. The mechanism may be related to loss of normal atrial systolic function, dysregulation of ventricular rhythm.

## Data Availability

The raw data supporting the conclusions of this article will be made available by the authors, without undue reservation.
